# Periodontal disease and some adverse perinatal outcomes in a cohort of low risk pregnant women

**DOI:** 10.1186/1742-4755-7-29

**Published:** 2010-11-03

**Authors:** Marianna Vogt, Antonio W Sallum, Jose G Cecatti, Sirlei S Morais

**Affiliations:** 1 Department of Prothesis and Periodontology. School of Odontology of Piracicaba, University of Campinas, Campinas, Brazil; 2 Department of Obstetrics and Gynecology. School of Medical Sciences, University of Campinas, Campinas, Brazil

## Abstract

**Objective:**

To evaluate the association of periodontal disease (PD) in pregnancy with some adverse perinatal outcomes.

**Method:**

This cohort study included 327 pregnant women divided in groups with or without PD. Indexes of plaque and gingival bleeding on probing, probing pocket depth, clinical attachment level and gingival recession were evaluated at one periodontal examination below 32 weeks of gestation. The rates of preterm birth (PTB), low birth weight (LBW), small for gestational age (SGA) neonates and prelabor rupture of membranes (PROM) were evaluated using Risk Ratios (95%CI) and Population Attributable Risk Fractions.

**Results:**

PD was associated with a higher risk of PTB (RR**_adj. _**3.47 95%CI 1.62-7.43), LBW (RR**_adj. _**2.93 95%CI 1.36-6.34) and PROM (RR**_adj. _**2.48 95%CI 1.35-4.56), but not with SGA neonates (RR 2.38 95%CI 0.93 - 6.10).

**Conclusions:**

PD was a risk factor for PT, LBW and PROM among Brazilian low risk pregnant women.

## Background

Through the last 12 years, periodontal disease (PD) has been suggested to be associated with preterm, low birth weight (PTB/LBW), and small for gestational age neonates (SGA), which are associated with higher risk of perinatal mortality, mortality in the first year of life, development of health problems during childhood (neurological, respiratory, gastrointestinal and cardiovascular), and risk of diseases during adulthood [1]. Now there is a debate that will be fomented by the clinical intervention experiments to determine if PD is a causal factor of those pregnancy adverse outcomes.

Maternal infections represent one of the most important risk factors for preterm birth (PTB). In 1996, a case-control study by Offenbacher *et al. *[2], suggested that PD can be associated with PTB/LBW through the same mechanisms as the other maternal infections, acting as a reservoir of gram-negative anaerobic microorganisms and their products, such as lipopolysaccharides and endotoxins, besides a large amount of inflammatory mediators, observed in the amniotic fluid or chorioamniotic membranes through hematogenous transportation. The inflammatory cytokines, such as IL-1β (interleukin-1β), IL-6 (interleukin 6), PGE_2 _(prostaglandin E_2_) and TNF-α (tumor necrosis factor) are related to the onset of labor, and can reach a critical level, inducing a tenderness of the uterine muscles, stimulating uterine contraction and cervical dilation, triggering PTB [3,4].

Epidemiological studies carried out in the last 15 years have demonstrated that women with poor periodontal health present higher risk of having PTB/LBW or SGA infants, compared to those with good periodontal health [2,4-8]. On the other hand, some studies have showing a modest association between PD and adverse pregnancy outcomes [9], or no association at all. [10-12]. Based on this concept, a number of intervention studies were carried out in an attempt to decrease adverse outcomes in pregnancy and the rate of PTB/LBW in populations after the treatment of PD, and concluded that PD seems to be an independent risk for PTB/LBW and that periodontal therapy in pregnant women with PD significantly reduces its occurrence [4,13,14]. Although all suggested this relationship, recent randomized controlled trials on the treatment of PD during pregnancy did not find any decrease in the rates of PTB, LBW or fetal growth restriction [15,16]. These differences could be explained by a lack of power due to a small sample size, by inadequate adjustment for confounders or mainly by distinct definitions of periodontitis [17].

In two Brazilian studies, a significant risk was observed between LBW and PD [18,19], but in the others, no association of periodontal infection and PTB/LBW was observed [20,21].

In spite of medical improvements and public health interventions in order to reduce PTB, they have increased in the last two decades and account for 6% to 15% of total deliveries, depending on the population studied. Moreover, a large proportion (approximately 70%) of PTB/LBW still has no known etiology, and, consequently, the identification of their risk factors seems important for the development of specific strategies for reducing their occurrence [22]. Thus, the purpose of this study is to evaluate the relationship between PD and some adverse perinatal outcomes in a Brazilian low risk pregnant women cohort and to assess if other clinical, habit and sociodemographic factors are also associated.

## Materials and methods

### Study Design

This is a contemporary cohort study that evaluates the relationship between PD and preterm birth, low birth weight, prelabor rupture of membranes and small for gestational age neonates, performed with low risk pregnant women (absence of severe systemic pathological conditions which could characterize high risk pregnancy: diabetes, severe hypertension, other chronic disease that already are a risk factors for the adverse outcomes) receiving prenatal care at the maternity of the University of Campinas, Brazil, who voluntarily agreed to participate in the study after signing an informed consent form. The study was approved by the Institutional Review Board.

### Study Population

Initially 334 pregnant women were included, aged 18 to 42, but seven cases were excluded: five due to spontaneous abortion, one due to unsuspected twin pregnancy and one due to fetal death, totalizing 327 at the final sample. Each participant underwent a single periodontal examination on the day of a scheduled prenatal visit, between February 2004 and August 2005.

### Inclusion of Subjects

Inclusion criteria were: gestational age ≤ 32 weeks and low risk. Women carrying twins, with a greater risk of preterm and/or low birth-weight (cervical incompetence, prior cervical surgery), with a previous preterm and with two or more Cesarean sections were excluded from the study.

Women attending the prenatal outpatient clinic were interviewed by a nurse during the educational support group meetings. The nurse briefly explained the objectives of the study and the procedures involved. Then they were referred directly to the odontological clinic to receive additional information and sign the informed consent form. Immediately afterwards, a questionnaire was filled out for collecting socio-demographic, habit and gestational variables and the periodontal examination was done.

### Periodontal Examination

It was carried out once during pregnancy before 32 weeks of gestation. The data were recorded on a clinical record form with a complete clinical and periodontal description of all the teeth including third molars. Oral hygiene status was assessed as the percentage of surfaces with plaque, by the dichotomous plaque index (presence or absence of plaque) (PI) [23]. Probing pocket depth (PPD: measurement from the gingival margin to the total probing depth), gingival recession (GR: measurement from the cemento-enamel junction to the gingival margin) and clinical attachment level (CAL: measurement from the cemento-enamel junction to the total probing depth) were evaluated at four tooth surfaces (mesial, buccal, distal and lingual) using a Williams periodontal probe. The greatest clinical measurement of each surface was registered. Bleeding on probing (BOP) was assessed during and recorded after PPD was measured, by the dichotomous index (presence or absence of bleeding), and was expressed as the percentage of surfaces showing bleeding [24].

The examinations were carried out by the same trained periodontist with experience in the field, and an assistant who provided technical support and who filled the data collection forms. The calibration of the exam with another independent professional evaluation or intra-examiner reliability was not performed due to complaints of the women on the length of time to be spent on that, taking into account the periodontal examination was performed at the same day, just before the prenatal visit. Although this could represent a possible limitation of the study, the procedure was performed with other patients out of the study.

### Criteria of Periodontal Diagnosis

The presence of 4 or more teeth showing at least one site with 4 mm of PPD and clinical attachment loss at the same site, with BOP, was diagnosed as periodontal disease (PD). These criteria were operationally selected for the clinical definition of pregnant women who positively and unequivocally exhibited PD specifically for this study. Therefore two groups of pregnant women were then defined with the purpose to associate them with the perinatal outcomes: one with PD and other without PD. In order to conduct a more accurate evaluation of the characteristics of PD in this population, the extension of the disease was also classified as follows: P1: at least four teeth with PPD and CAL of 4-6 mm; P2: at least four teeth with PPD and CAL of 7-9 mm; and P3: at least four teeth with PPD and CAL of 10 mm. Among the women classified as without PD for this study, those that had BOP in more than 25% of sites were classified as having only gingivitis in some sites, and when it was ≤ 25%, they were classified as having healthy periodontal status [3].

### Perinatal Outcome Variables

The pregnancy outcome variables were preterm birth (PTB-defined as occurring before 37 weeks of gestational age), low birth weight (LBW-defined as birthweight below 2500 g), newborn small for gestational age (SGA-defined as birthweight below the 10^th ^percentile of the normal curve of birthweight according to gestational age using the Lubchenco curve as reference) and prelabor rupture of membranes (PROM-rupture of the membranes occurring before the onset of labor, diagnosed clinically, with increased vaginal pH and decreased amount of amniotic fluid confirmed by ultrasound scan), obtained from the pregnant women's files and clinical records (or by telephone contact in the few cases the woman gave birth in another hospital), after the estimated date for delivery.

### Data Collection and Control Variables

Informations were collected from the pregnant women's files and at the time of the exam, by means of a questionnaire, in order to identify some variables that could modify the relationship between the periodontal status during pregnancy and perinatal outcomes. These variables were: socio-demographic variables (age, parity, race/color, years of schooling, marital status, body mass index-BMI-estimated with the pre pregnancy weight, and any systemic diseases), habit variables (smoking and alcohol consumption) and gestacional variables (number of prenatal visits, bacterial vaginosis, vaginal delivery and the newborn Apgar scores at the first and fifth minute of life).

### Statistical Analysis

When each case was finished, with information on delivery and perinatal outcomes available, the form was checked for completeness and correctness. Then the information was entered to feed a computer database specifically prepared for this study. Consistency tests were then performed to identify errors which are corrected after checking the correspondent information in the clinical records. For the analysis, initially frequency distribution of the socio-demographic, habit and gestational variables among both groups were performed, with the differences between groups evaluated by χ^2 ^or Fisher Exact tests.

Afterwards the univariate and multivariate analyses (variables selected by the Stepwise method) were carried out, with the incidence of the perinatal outcome variables assessed in both groups, respectively estimating the crude and adjusted Risk Ratio and its respective 95%CI. For each model performed for multivariate analysis for each main outcome, besides the group all other predictors entered the model. Additionally the Population Attributable Risk Fraction (PARF) was also calculated for the main outcomes. The level of significance was established at 5%, and Epi Info 6.0 and SAS were used for the statistical analysis procedures.

## Results

Among the 334 pregnant women evaluated and followed until delivery, a total of 327 had their deliveries and the pregnancy outcomes were recorded. Among these, 156 had PD and 171 had been classified as without PD, what represents a 47% prevalence of exposure (Table 1). In general, the women presented similar socio-demographic characteristics and habits in both groups. Likewise, the groups did not present significant differences regarding the number of prenatal visits, diagnosis of bacterial vaginosis during pregnancy, vaginal delivery, nor did they have different Apgar scores at the first and fifth minute of life (Table 2).

**Table 1:** Percent distribution of pregnant women according to the periodontal status based on clinical attachment level (CAL)

Periodontal Status	N	%
**Without PD**	177	53
**With PD**	157	47
**P1 (CAL 4-6 mm)**	133	39.8
**P2 (CAL 7-9 mm)**	20	6.0
**P3 (CAL ≥ 10 mm)**	4	1.2
**Total**	**334**	**100.0**

**Table 2:** Distribution of pregnant women by a number of control and gestational variables (socio-demographic and habit variables), according to the presence of periodontal disease

	Periodontal disease		
		
Characteristics	With	Without	p*
**Control variables**			
Age < 25 years old	53 (34.0%)	70 (40.9%)	0.194
White women	80 (51.3%)	93 (54.4%)	0.570
Schooling up to primary level	98 (62.8%)	92 (53.8%)	0.098
Stable partner	146 (93.6%)	164 (95.9%)	0.229
Nuliparity	61 (39.1%)	77 (45.0%)	0.278
Smoking	19 (12.2%)	13 (7.6%)	0.164
Alcohol	11 (7.0%)	12 (7.0%)	0.990
Systemic disease	31 (19.9%)	37 (21.6%)	0.690
Normal BMI (19,8-26)	81 (51.9%)	99 (57.9%)	0.278
**Gestational variables**			
Number of prenatal visits			0.780
1-5	20 (12.8%)	19 (11.1%)	
6-10	101 (64.7%)	109 (63.7%)	
11 +	34 (21.8%)	42 (24.6%)	
Bacterial vaginosis	36 (23.0%)	29 (17.0%)	0.166
Vaginal delivery	99 (63.5%)	99 (57.9%)	0.303
Apgar 1^st ^min < 7 **	14 (9.0%)	21 (12.3%)	0.367
Apgar 5^th ^min < 7 ** #	2 (1.3%)	4 (2.3%)	0.384
**Total**	**156 (100%)**	**171 (100%)**	

* χ^2 ^test

** lack of information on Apgar score for newborns who were born in other hospitals

# Fisher exact test

The incidence of PTB was 12.2% in the group with PD and 6.4% in group without PD, but this difference was not statistically significant. The incidence of LBW and SGA newborns were also similar between both groups in the univariate analysis. Finally, 24.4% incidence of PROM in the group with PD compared to the 9.4% in the group without PD was the only statistically significant difference found in the univariate analysis, representing a two and a half times higher risk of PROM for those with PD (Table 3).

**Table 3:** Distribution of pregnant women by the occurrence of perinatal outcomes (preterm birth-PTB, low birth weight-LBW, small for gestational age babies-SGA and prelabor rupture of membranes-PROM), their RR and PARF, according to the presence of periodontal disease

	Periodontal Disease		RR (95% CI)	PARF (%)
			
Perinatal outcomes	With	Without		
**Preterm birth**	19 (12.2%)	11 (6.4%)	1.89 (0.93 - 3.85)	29.1
**LBW**	18 (11.5%)	12 (7.0%)	1.64 (0.82 - 3.30)	22.9
**SGA**	13 (8.3%)	6 (3.5%)	2.38 (0.93 - 6.10)	38.9
**PROM**	38 (24.4%)	16 (9.4%)	2.62 (1.52 - 4.51)	42.4
**Total**	**156 (100%)**	**171 (100%)**		

RR: risk ratio

PARF: Population Attributable Risk Fraction

The calculation of the population attributable risk fraction (PARF) for each of these conditions showed that, for this population, at least in theory, the percentage of cases of PTB that would be avoided with the exclusion of the PD is 29%, as well as 42% of PROM (Table 3).

When the multivariate regression analysis model including the other predictor variables was fitted, PD was found to be independently and statistically associated also with PTB and LBW, besides PROM, with risk ratio adjusted by the other statistically higher factors (Table 4).

**Table 4:** Variables statistically associated with adverse perinatal outcomes and their respective RR_adj. _(95%CI) by the multivariate analysis

Perinatal outcomes	Selected Variable *	RR_adj. _(95% CI)
Preterm birth*	Periodontal disease	3.47 (1.62 - 7.43)
	Nr. Antenatal visits ≤ 5	6.18 (2.88 - 13.23)

Low birth weight*	Periodontal disease	2.93 (1.36 - 6.34)
	Nr. Antenatal visits ≤ 5	5.23 (2.42 - 11.3)

**Newborn SGA**	No selected variable	-

**Prelabor Rupture of Membranes**	Periodontal Disease	2.48 (1.35 - 4.56)

* Selection of variables through the Stepwise method

RR_adj _= Risk ratio adjusted by multivariate analysis

## Discussion

The association between PD and the adverse outcomes of pregnancy that were found in this study are consistent with those in other studies [2,4,5,7,18]. They differ from outcomes of a study [25] which used the Community Periodontal Index of Treatment Needs [26], a partial periodontal evaluation that does not include important periodontal clinical parameters, such as CAL, and did not find any evidence of this association. We observed a difference between the conclusions of the studies due to distinct definitions of periodontitis [17] and also the large variation of the populations studied. For example, the multi-ethnic population of a study in England [25], basically white natives and Bengali immigrants, with peculiar habits such as chewing tobacco, very different from the black population studied by the North-American investigators [2,5], from the Jordan women [7], from the Pakistan women [9], and from the Brazilian low income women of the current study. It is also possible that unknown genetic, stress and environmental factors can influence differences in the outcomes [27].

There is also a difficulty in comparing these studies and carrying out a meta-analysis [28], since the investigators do not use a standardized measurement to evaluate PD, such as CAL, PPD and other periodontal parameters, in addition to the different indexes, such as CPITN, Russel, PSR (Periodontal Screening and Recording), and other less direct measurements, such as antibodies to periodontal pathogens [17,27].

Knowing that PD and PTB have common risk factors, such as smoking and diabetes *melittus*, we can highlight the need for a good design of the studies, so that risk factors, as well as sociodemographic and habit variables, are controlled and adjusted, in order to scientifically achieve real and significant outcomes. That is what this study has intended to do, mainly with this cohort approach, which identified that, for the population studied, PD was statistically associated with PTB, LBW and PROM, after controlling all the other variables as potential confounders.

The prevalence of 47% of PD found in the current study reflect moderate PD forms (39.8% of P1 - 133 cases), severe PD forms (7.2% of P2 and P3 - 24 cases) as well as the 4 cases of P3 should be classified as aggressive forms of periodontitis [29]. Its association with adverse perinatal outcomes was also identified. Our intention was really to isolate and observe the effect of periodontal infection, specially these ones with attachment loss, on perinatal outcomes. As it is known, patient who is considered to be at risk for severe periodontitis has an inflammatory indication that is different from that of a low risk patient [3]. The outcomes of recent study [30], which included only women with gingivitis, without any periodontal attachment loss, indicated that the treatment of gingivitis associated with pregnancy reduced the incidence of PTB/LBW in up to 3.26 times. An original study [8] pointed out the total inflammatory burden from the oral cavity, including the presence of dental calculus and mouth ulcers, besides gingival BOP and PPD, all associated with preterm birth. Under this point of view, we have therefore pointed out the importance of the role of the inflammatory profile of each patient, besides the degree of periodontal infection she may have, or the bacterial species involved on that [31].

The current prospective study didn't find a relationship between PD and SGA neonates; the results of retrospective study [9] pointed out that moderate and severe PD early in pregnancy is associated with delivery of a SGA infant, as a result of fetal grow restriction. The present consensus is that there seems to be an association between PD and PTB/LBW/SGA, but it is still not clear whether the PD really represents a factor which causes the adverse outcomes of pregnancy [22]. A preliminary evidence that has been found so far suggests that the periodontal treatment during pregnancy is safe and can reduce undesirables outcomes, as demonstrated by a new metaanalysis of randomized trials [28] besides several limitations on that. The conflicting results show that many epidemiological studies and mainly that of intervention are still necessary to validate this association and determine whether it is a cause and how great a risk it represents, since its big social impact [32]. Actually, there still does not exist a large systematic review of the international scientific literature which describes the outcomes of large clinical trials to clarify such points.

Recently the results of one of these big trials came out [15], but the findings were completely different from what they were expected. They did not find any association between treatment of PD during pregnancy with significant reduction in the occurrence of PTB, LBW or fetal growth restriction. After that, that clinical and birth outcomes were used to another study which showed that PD progression was not associated with an increased risk for PTB or LBW infant [11]. However, the most important clinical implication observed was that periodontal treatment during pregnancy is efficient to control the disease process, which seems, based on studies' results and on these of current study, to be associated with negative birth outcomes, although it is not necessarily causal. Based on that, our findings are important because showed this association besides the need of the oral health program during prenatal care for this studied Brazilian population. In addition, the knowledge about the relationship between oral and systemic disorders, like the undesirables perinatal outcomes focused here, provides us special findings that have clinical implications. The most important of them is the identification of shared risk factors between oral and this systemic process.

Now, we have a range of knowledge to assist our patients, controlling their risk factors with the purpose to control their oral health. But, as much as the risk factors for PD coincide with some of that for PTB, LBW and SGA (like smoking, diabetes *melittus*, stress), when we control a risk complex, we control the other. In this case, since the risk factors for PD are also associated with adverse perinatal outcomes, the causality should not be a determinant factor to the treatment. Therefore, the evaluation of the risk and the control of PD must become a central function of the odontological practice, to improve the quality of life and well being of the expectant mother and her baby.

Although there is not so much strong high level scientific evidence on the influence of PD on the perinatal outcomes of pregnancy, in general the outcomes are usually similar and consistent with those of this present study, showing that bad oral conditions could play a significant role in determining adverse perinatal outcomes, such as those this study focused on [2,4-7]. This can be made clear by the magnitude of the risk ratio found by this and other studies in this area. New epidemiologic and intervention studies are necessary to better evaluate whether this association really represents a relationship of cause and effect, or if PD is only an additional factor for PTB/LBW, which is of considerable social importance.

Some limitations of the current study could of course be pointed out, perhaps including the limited sample size and also the periodontal examination being performed in different gestational ages, with no control of its evolution during pregnancy. However, we judge that one original aspect of this study is related to the calculation of the population attributable risk fraction (PARF) for each of the adverse perinatal conditions [33]. Perhaps this may help to supply a more clearly understandable measurement, in the context of public health, of the importance of intervention studies that could clarify the role of PD and its treatment in the perinatal outcomes. If an oral health program could really be made in populations with high incidence of PD, eliminating it completely, an important proportion of these adverse perinatal conditions could probably be avoided, at least in theory. Even understanding that these adverse perinatal outcomes are derived from multiple causes, this is a challenge for the future when a definitive association between PD and adverse perinatal outcomes could be available.

## Disclosure of interests

The authors declare that they have no competing interests.

## Authors' contributions

MV and AWS had the original idea for the study. MV and JGC wrote the first version of the proposal. AWS got the grant for implementation of the study. MV and JGC were responsible for data collection. SSM, MV, and JGC were responsible for data analysis. MV and JGC wrote the first draft of the paper and then all the others gave important inputs and suggestions for interpretation and improvement of the manuscript. All authors have read the final version of the article and agreed with it.
